# Timing of Tracheostomy in ICU Patients: A Systematic Review and Meta-Analysis of Randomized Controlled Trials

**DOI:** 10.3390/life14091165

**Published:** 2024-09-14

**Authors:** Raffaele Merola, Carmine Iacovazzo, Stefania Troise, Annachiara Marra, Antonella Formichella, Giuseppe Servillo, Maria Vargas

**Affiliations:** 1 Anesthesia and Intensive Care Medicine, Department of Neurosciences, Reproductive and Odontostomatological Sciences, University of Naples Federico II, 80131 Naples, Italy; carmine.iacovazzo@unina.it (C.I.); dottmarraannachiara@gmail.com (A.M.); antonellaformichella@yahoo.it (A.F.); maria.vargas@unina.it (G.S.); vargas.maria82@gmail.com (M.V.); 2 Maxillofacial Surgery Unit, Department of Neurosciences, Reproductive and Odontostomatological Sciences, University of Naples Federico II, 80131 Naples, Italy; stefania.troise@unina.it

**Keywords:** early tracheostomy, intensive care unit, critically ill patients, mechanical ventilation, trial sequential analysis

## Abstract

**Background:** The ideal timing for tracheostomy in critically ill patients is still debated. This systematic review and meta-analysis examined whether early tracheostomy improves clinical outcomes compared to late tracheostomy or prolonged intubation in critically ill patients on mechanical ventilation. **Methods:** We conducted a comprehensive search of randomized controlled trials (RCTs) assessing the risk of clinical outcomes in intensive care unit (ICU) patients who underwent early (within 7–10 days of intubation) versus late tracheostomy or prolonged intubation. Databases searched included PubMed, Embase, and the Cochrane Library up to June 2023. The primary outcome evaluated was mortality, while secondary outcomes included the incidence of ventilator-associated pneumonia (VAP), ICU length of stay, and duration of mechanical ventilation. No language restriction was applied. Eligible studies were RCTs comparing early to late tracheostomy or prolonged intubation in critically ill patients that reported on mortality. The risk of bias was evaluated using the Cochrane Risk of Bias Tool for RCTs, and evidence certainty was assessed via the GRADE approach. **Results:** This systematic review and meta-analysis included 19 RCTs, covering 3586 critically ill patients. Early tracheostomy modestly decreased mortality compared to the control (RR −0.1511 [95% CI: −0.2951 to −0.0070], *p* = 0.0398). It also reduced ICU length of stay (SMD −0.6237 [95% CI: −0.9526 to −0.2948], *p* = 0.0002) and the duration of mechanical ventilation compared to late tracheostomy (SMD −0.3887 [95% CI: −0.7726 to −0.0048], *p* = 0.0472). However, early tracheostomy did not significantly reduce the duration of mechanical ventilation compared to prolonged intubation (SMD −0.1192 [95% CI: −0.2986 to 0.0601], *p* = 0.1927) or affect VAP incidence (RR −0.0986 [95% CI: −0.2272 to 0.0299], *p* = 0.1327). Trial sequential analysis (TSA) for each outcome indicated that additional trials are needed for conclusive evidence. **Conclusions:** Early tracheostomy appears to offer some benefits across all considered clinical outcomes when compared to late tracheostomy and prolonged intubation.

## 1. Introduction

Tracheostomy is frequently performed in critically ill patients requiring prolonged mechanical ventilation [1]. Tracheostomy is thought to reduce the incidence of ventilator-associated pneumonia (VAP), duration of mechanical ventilation, length of ICU stay, and risk of death. This is due in part to the reduced need for sedation, increased patient comfort, improved communication skills and oral hygiene, and the ability to swallow and drink/feed orally [2,3,4,5]. However, tracheostomy also carries risks such as bleeding, wound infection, tracheal stenosis, accidental displacement, and occasionally death [6,7]. Determining the optimal timing of tracheostomy in critically ill patients undergoing mechanical ventilation is critical to improve clinical outcomes. In 1989, a consensus conference recommended that tracheostomy should be performed 3 weeks after endotracheal intubation [8]. Despite this, there is still a debate about the best time to perform tracheostomy in critically ill patients. Several studies have evaluated the efficacy and safety of early versus late tracheostomy in these patients, with inconsistent results [9,10,11,12,13]. Previous systematic reviews and meta-analyses have also shown a differential impact of early versus late tracheostomy on clinical outcomes [14,15,16,17]. A recent meta-analysis of RCTs found that early tracheostomy reduced the length of ICU stay and duration of mechanical ventilation but did not significantly reduce the risk of short-term mortality and VAP [18]. Another study by Quinn et al. used a Bayesian random-effects meta-analysis approach and concluded that early tracheostomy offers some benefits across all clinical outcomes compared with delayed tracheostomy [19]. In this systematic review with meta-analysis and trial sequential analysis (TSA), we aimed to evaluate whether early tracheostomy, compared with delayed tracheostomy or prolonged intubation, can improve clinical outcomes in critically ill patients receiving mechanical ventilation. The primary outcome measured was mortality, while secondary outcomes included the incidence of ventilator-associated pneumonia (VAP), length of intensive care unit stay, and duration of mechanical ventilation.

## 2. Materials and Methods

### 2.1. Data Sources and Search Strategy

This systematic review and meta-analysis was conducted according to the Preferred Reporting Items for Systematic Reviews and Meta-Analyses (PRISMA) guidelines. This study was not registered in any database. We conducted a comprehensive search for all randomized controlled trials (RCTs) comparing the risk of clinical outcomes in ICU patients who underwent early tracheostomy (within 7–10 days of intubation) versus late tracheostomy (after 7–10 days of intubation) or prolonged intubation. The search encompassed PubMed, Embase, and the Cochrane Library from their inception until June 2023. Both subject headings and text-word terms were used to identify relevant articles. The search terms included “tracheotomy” OR “tracheostomy” OR “intratracheal intubation” AND (“mechanical ventilation” OR “artificial respiration”) AND “randomized controlled trials”. We did not impose any language restrictions on the search. Furthermore, we cross-referenced the original articles and reviews to identify additional relevant studies.

The inclusion criteria focused on RCTs that compared early tracheostomy to late tracheostomy or prolonged intubation in ICU patients and reported clinical outcomes of interest. Case reports, observational studies, and cross-over studies were excluded from the review. Participants included in the analysis were critically ill patients of any age and sex.

### 2.2. Data Extraction and Quality Assessment

Two pairs of independent reviewers (RM, ST and CI, MV) performed the initial selection by reviewing titles and abstracts. Each reviewer independently evaluated citations to identify additional RCTs for inclusion in the analysis. For detailed evaluation, a full-text copy of all potentially relevant studies was obtained. Data from each study, including sample size, timing of tracheostomy, and outcomes of interest, were independently extracted by paired reviewers (RM, ST and CI, MV) using a pre-standardized data extraction form. A separate pair of reviewers (AM, AF) conducted a full-text evaluation without knowing the authors, institutional affiliations, journal, or publication date. Another reviewer (GS) checked the accuracy of the data extracted from the publications.

### 2.3. Outcome Measures

The primary outcome was mortality. Secondary outcomes included the incidence of VAP, ICU length of stay, and duration of mechanical ventilation. We planned a priori to conduct subgroup analyses on both primary and secondary outcomes to evaluate the impact of early tracheostomy versus late tracheostomy and early tracheostomy versus prolonged intubation when possible.

### 2.4. Qualitative Analysis

Two reviewers (MV, RM) independently assessed the quality of the studies. Any disagreements were resolved by consensus or consultation with a third reviewer (CI), if necessary. Risk of bias was assessed using the Cochrane Risk of Bias Tool for RCTs. Certainty of evidence was assessed using the GRADE approach. Within GRADE, the body of evidence for each outcome was assessed according to different domains that could reduce or increase the certainty of evidence. Factors that may reduce certainty include the risk of bias (study limitations), inconsistency (unexplained heterogeneity among study results), indirectness (applicability or generalizability to the research question), imprecision (the confidence in estimating an effect to support a particular decision) or publication bias (selective publication of studies). The certainty of the evidence can be strengthened if the following considerations are present: high or very high effect size, evidence of a dose-response gradient, or residual opposite confounding. GRADE summary of findings and tables were developed with GRADEpro GDT software (McMaster University, 2015. Developed by Evidence Prime, Inc., Krakow, Poland. Available at: https://gradepro.org/, accessed on 13 September 2023).

### 2.5. Quantitative Analysis

The analysis used the logarithmic hazard ratio as the outcome measure, fitting a random effects model to the data. The DerSimonian-Laird estimator was used to estimate the amount of heterogeneity (tau^2^). For dichotomous outcomes, we used the risk ratio (RR), while for continuous outcomes, the standardized mean difference (SMD) was applied. In addition to the tau^2^ estimation, we reported Cochran’s Q test for heterogeneity and the I^2^ statistic. The Q test, based on a chi-square distribution, indicates greater variation between studies than within subjects within a study if the probability is high. The I^2^ statistic quantifies heterogeneity as less than 25% indicating low heterogeneity, 25% to 50% indicating moderate heterogeneity, and over 50% indicating high heterogeneity. When some degree of heterogeneity was detected (tau^2^ > 0), a prediction interval for the actual results was provided. To identify outliers and influential studies, we used studentized residuals and Cook’s distances. Studies with a studentized residual exceeding the 100 × (1 − 0.05/(2 × k)) percentile of a standard normal distribution were considered potential outliers, applying a Bonferroni correction with a two-sided alpha of 0.05 for k studies in the meta-analysis. Studies with a Cook’s distance greater than the median plus six times the interquartile range of the Cook’s distances were deemed influential. Funnel plot asymmetry was assessed using the rank correlation test and the regression test, with the standard error of the observed outcomes as predictors. Analyses were conducted with Jamovi (version 2.3.18).

Trial sequential analysis (TSA) depends on the required information size (RIS) quantification. TSA was performed using TSA 0.9 beta software if more than five trials were included. The RIS was estimated using relative risk reduction and heterogeneity-adjusted information size for dichotomous outcomes. Results were confirmed as true positives if the cumulative Z-curve surpassed the Lan-DeMets trial sequential monitoring boundary or reached the RIS above the conventional significance level line (Z = 1.96). Conversely, results were confirmed as true negatives if the cumulative Z-curve reached the futility boundary or the RIS below the conventional significance level line (Z = 1.96). TSA-adjusted 95% confidence intervals (CIs) were also presented.

## 3. Results

**Figure 1:**
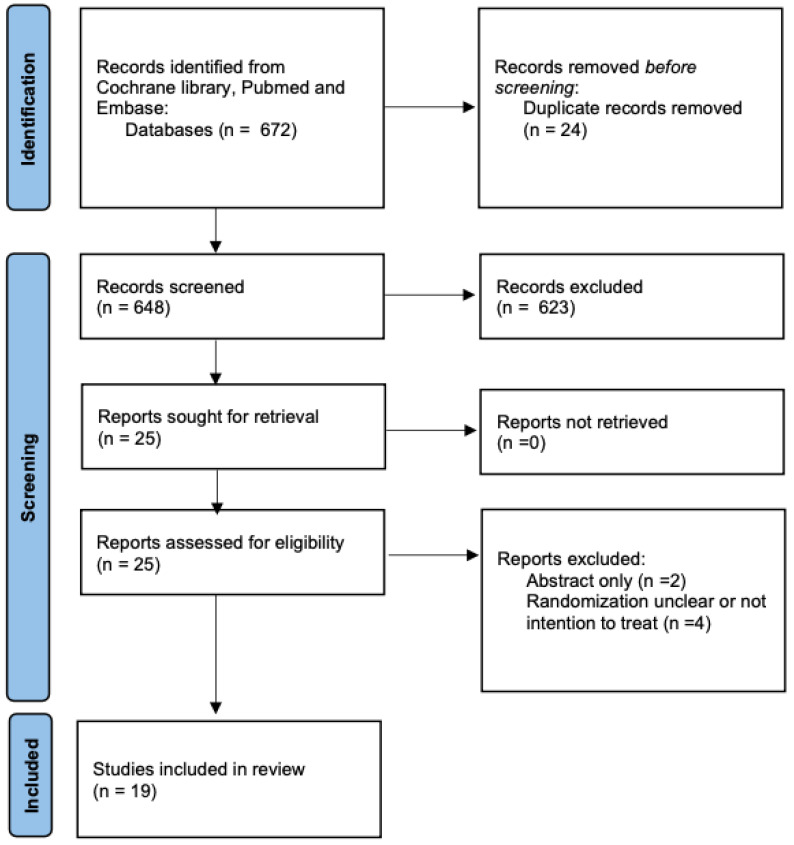
PRISMA 2020 flow diagram for new systematic reviews.

The electronic search yielded 672 potential articles. After removing duplicates, we screened 648 articles by title and abstract for eligibility. From these, we selected twenty-five trials for full-text assessment. Six trials were excluded for not meeting our inclusion criteria. Ultimately, nineteen trials, evaluating 3586 critically ill patients, were included in our review [9,10,11,12,13,20,21,22,23,24,25,26,27,28,29,30,31,32,33]. The details of the screening process are illustrated in Figure 1. Table 1 presents the baseline characteristics of the included studies. All included studies demonstrated a low risk of bias, as shown in Table 2.

### 3.1. Mortality

**Figure 2:**
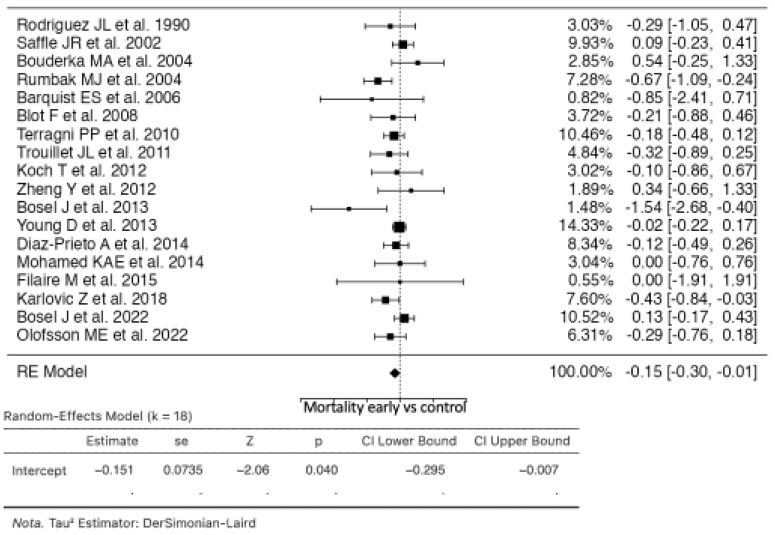
Forest plot of estimated risk ratio comparing risk of mortality in the early tracheostomy vs. control group [10,12,13,20,21,22,23,24,25,26,27,28,29,30,32,33].

Eighteen studies were included in the analysis comparing early tracheostomy to the control group (Figure 2). The estimated average risk ratio (RR) based on the random effects model was −0.1511 (95% CI: −0.2951 to −0.0070), indicating a significant difference from zero (z = −2.0555, *p* = 0.0398). Although the Q-test for heterogeneity was not significant, suggesting that some heterogeneity may still be present in the true outcomes (Q(17) = 25.9367, *p* = 0.0756, tau^2^ = 0.0278, I^2^ = 34.4559%), Cook’s distances indicated that none of the studies were overly influential. The rank correlation and regression tests did not indicate any funnel plot asymmetry (*p* = 0.6540 and *p* = 0.3085, respectively) [9,10,11,12,13,20,21,22,23,24,25,26,27,28,29,30,32,33].

Fourteen studies were included in the analysis comparing early tracheostomy to late tracheostomy. The estimated average RR based on the random effects model was −0.1670 (95% CI: −0.3273 to −0.0067) (Supplementary Materials), which was significantly different from zero (z = −2.0424, *p* = 0.0411). The Q-test indicated heterogeneity in the true outcomes (Q(13) = 22.6967, *p* = 0.0455, tau^2^ = 0.0334, I^2^ = 42.7230%). Funnel plot asymmetry was not suggested by the rank correlation or regression tests (*p* = 0.1572 and *p* = 0.0960, respectively) [10,11,12,13,20,21,23,25,26,27,28,30,32,33].

Three studies were included in the analysis comparing early tracheostomy to prolonged intubation. The estimated average RR based on the random effects model was 0.0936 (95% CI: −0.385 to 0.572) (Supplementary Materials), which did not significantly differ from zero (z = 0.384, *p* = 0.0701). The Q-test indicated heterogeneity in the true outcomes (Q(2) = 3.0455, *p* = 0.0455, tau^2^ = 0.0334, I^2^ = 42.7230%). Neither the rank correlation nor the regression test indicated any funnel plot asymmetry (*p* = 0.702 and *p* = 0.0960, respectively) [9,22,29].

### 3.2. Ventilator-Associated Pneumonia

**Figure 3:**
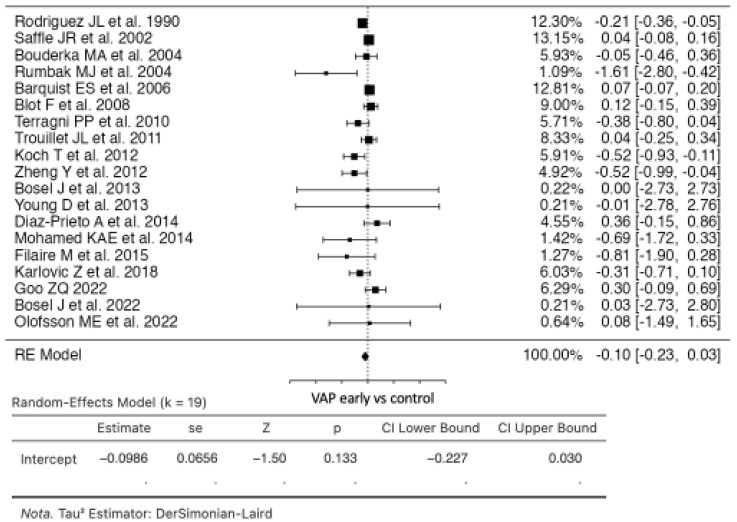
Forest plot of estimated risk ratio comparing risk of VAP in the early tracheostomy vs. control group [10,12,13,20,21,22,23,24,25,26,27,28,29,30,32,33].

In the analysis comparing early tracheostomy to the control group, nineteen studies were included (Figure 3). The estimated average risk ratio (RR) based on the random-effects model was −0.0986 (95% CI: −0.2272 to 0.0299). Therefore, the average outcome did not show a significant difference from zero (z = −1.5036, *p* = 0.1327). The Q-test indicated heterogeneity in the true outcomes (Q(18) = 39.1169, *p* = 0.0027, tau^2^ = 0.0289, I^2^ = 53.9841%). Funnel plot asymmetry was not indicated by either the rank correlation test or the regression test (*p* = 0.2983 and *p* = 0.0811, respectively) [9,10,11,12,13,20,21,22,23,24,25,26,27,28,29,30,31,32,33].

Twelve studies were included in the analysis comparing early tracheostomy to late tracheostomy. The estimated average RR based on the random effects model was −0.1461 (95% CI: −0.3129 to 0.0208) (Supplementary Materials). Thus, the average outcome did not significantly differ from zero (z = −1.7158, *p* = 0.0862). The Q-test indicated significant heterogeneity in the true outcomes (Q(11) = 35.4719, *p* = 0.0002, tau^2^ = 0.0417, I^2^ = 68.9895%). Neither the rank correlation nor the regression test suggested funnel plot asymmetry (*p* = 0.2496 and *p* = 0.0503, respectively) [10,11,13,20,21,23,25,27,28,30,31,33].

Four studies were included in the analysis comparing early tracheostomy to prolonged intubation. The estimated average RR based on the random effects model was 0.0353 (95% CI: −0.1414 to 0.2120). Therefore, the average outcome did not significantly differ from zero (z = 0.3916, *p* = 0.6953) (Supplementary Materials). The Q-test showed no significant heterogeneity in the true outcomes (Q(3) = 2.8670, *p* = 0.4126, tau^2^ = 0.0000, I^2^ = 0.0000%). Neither the rank correlation nor the regression test indicated any funnel plot asymmetry (*p* = 0.0833 and *p* = 0.0934, respectively) [9,22,24,29].

### 3.3. ICU Length of Stay

**Figure 4:**
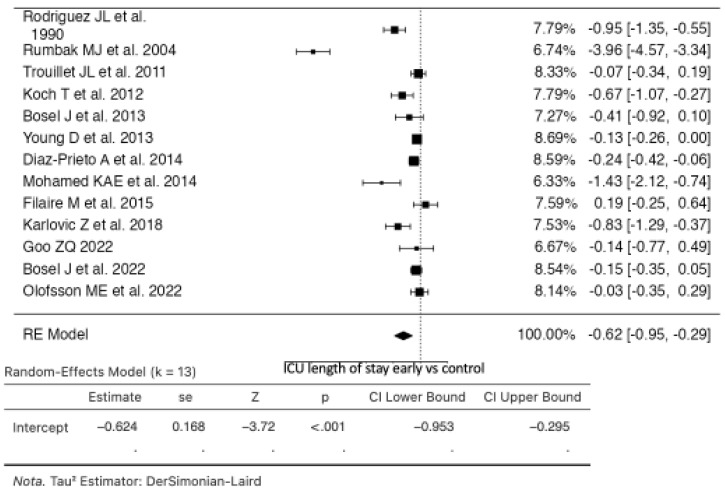
Forest plot of estimated standardized mean difference comparing ICU length of stay in the early tracheostomy vs. control group [10,12,20,24,25,26,27,28,29,30,31,32,33].

In the analysis comparing early tracheostomy to the control group, thirteen studies were included (Figure 4). The estimated average standardized mean difference (SMD) based on the random effects model was −0.6237 (95% CI: −0.9526 to −0.2948). Therefore, the average outcome differed significantly from zero (z = −3.7163, *p* = 0.0002). The Q-test indicated significant heterogeneity in the true outcomes (Q(12) = 183.7790, *p* < 0.0001, tau^2^ = 0.3195, I^2^ = 93.4704%) [10,12,20,24,25,26,27,28,29,30,31,32,33].

Upon examination of studentized residuals, one study [10] had a value larger than ±2.8905, suggesting it may be a potential outlier in this model. Cook’s distances also identified this study as potentially overly influential. The regression test indicated funnel plot asymmetry (*p* = 0.0006), though the rank correlation test did not (*p* = 0.0573).

Twelve studies were included in the analysis comparing early tracheostomy to late tracheostomy. The estimated average SMD based on the random-effects model was −0.6918 (95% CI: −1.0373 to −0.3462) (Supplementary Materials). Thus, the average outcome differed significantly from zero (z = −3.9237, *p* < 0.0001). The Q-test showed significant heterogeneity in the true outcomes (Q(11) = 179.2880, *p* < 0.0001, tau^2^ = 0.3267, I^2^ = 93.8646%) (Supplementary Materials) [10,12,20,24,25,26,27,28,30,31,32,33].

Upon examining studentized residuals, one study [10] had a value larger than ±2.8653, suggesting it may be a potential outlier. Cook’s distances also identified this study as potentially overly influential. Both the rank correlation and regression tests indicated potential funnel plot asymmetry (*p* = 0.0311 and *p* = 0.0002, respectively).

These findings suggest significant variability among studies in both comparisons, indicating caution in interpreting the pooled results due to the presence of outliers, potential influential studies, and funnel plot asymmetry.

### 3.4. Duration of Mechanical Ventilation

**Figure 5:**
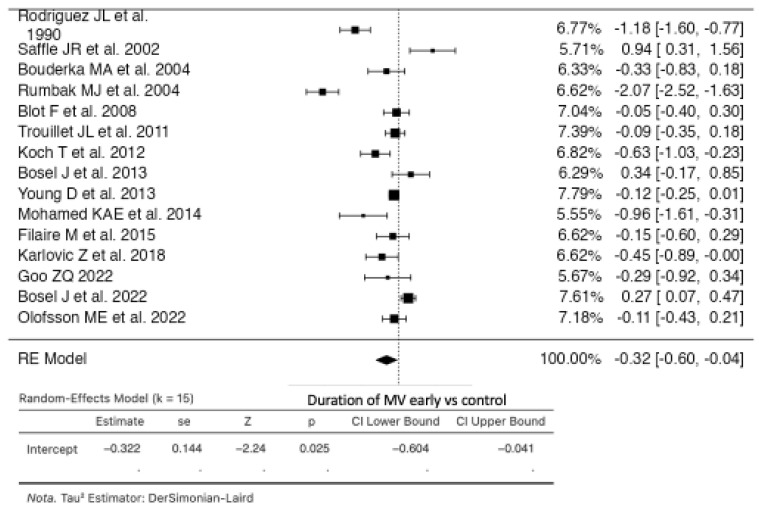
Forest plot of estimated standardized mean difference comparing duration of mechanical ventilation in the early tracheostomy vs. control group [10,12,20,24,25,26,27,28,29,30,31,32,33].

Fifteen studies were analyzed comparing early versus control groups (see Figure 5). Using a random-effects model, the estimated average Standardized Mean Difference (SMD) was −0.3225 (95% CI: −0.6040 to −0.0409). This indicates a statistically significant difference from zero (z = −2.2446, *p* = 0.0248). The Q-test suggested heterogeneous true outcomes (Q (14) = 142.3639, *p* < 0.0001, tau^2^ = 0.2605, I^2^ = 90.1660%). Examination of studentized residuals identified one study [10] as a potential outlier with a value exceeding ± 2.9352 in this model. According to Cook’s distances, this study may exert undue influence. Funnel plot asymmetry was not indicated by rank correlation (*p* = 0.3282) or regression tests (*p* = 0.6129) [9,10,12,20,21,22,24,25,26,28,29,30,31,32,33].

Eleven studies were included in the analysis comparing early versus late tracheostomy. The random-effects model estimated an average SMD of −0.3887 (95% CI: −0.7726 to −0.0048) (Supplementary Materials). This shows a statistically significant deviation from zero (z = −1.9845, *p* = 0.0472). Heterogeneity in true outcomes was indicated by the Q-test (Q (10) = 140.9760, *p* < 0.0001, tau^2^ = 0.3689, I^2^ = 92.9066%). One study [10] was identified as a potential outlier based on studentized residuals with a value exceeding ±2.8376. Cook’s distances did not identify any studies as overly influential. Neither rank correlation (*p* = 0.5423) nor regression tests (*p* = 0.7892) indicated funnel plot asymmetry [10,12,20,21,25,26,28,30,31,32,33].

Four studies were analyzed comparing early tracheostomy versus prolonged intubation. The random-effects model estimated an average SMD of −0.1192 (95% CI: −0.2986 to 0.0601) (Supplementary Materials). This did not show a statistically significant difference from zero (z = −1.3027, *p* = 0.1927). The Q-test indicated no significant heterogeneity in true outcomes (Q (3) = 0.8670, *p* = 0.8334, tau^2^ = 0.0000, I^2^ = 0.0000%). Funnel plot asymmetry was not indicated by rank correlation (*p* = 0.3333) or regression tests (*p* = 0.4783) [9,22,24,29].

### 3.5. Certainty of the Evidence Assessment

**Figure 6:**
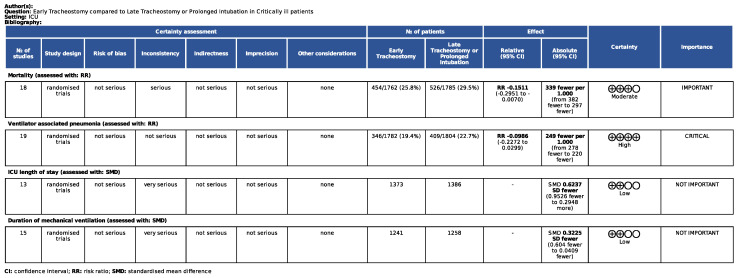
GRADE evidence profile for the considered outcomes.

Overall, evidence was qualified using GRADE for RCTs (Figure 6). High-quality evidence was found for VAP, moderate-quality evidence was found for mortality, and low-quality evidence was found for ICU length of stay and duration of mechanical ventilation.

### 3.6. Trial Sequential Analysis

**Figure 7:**
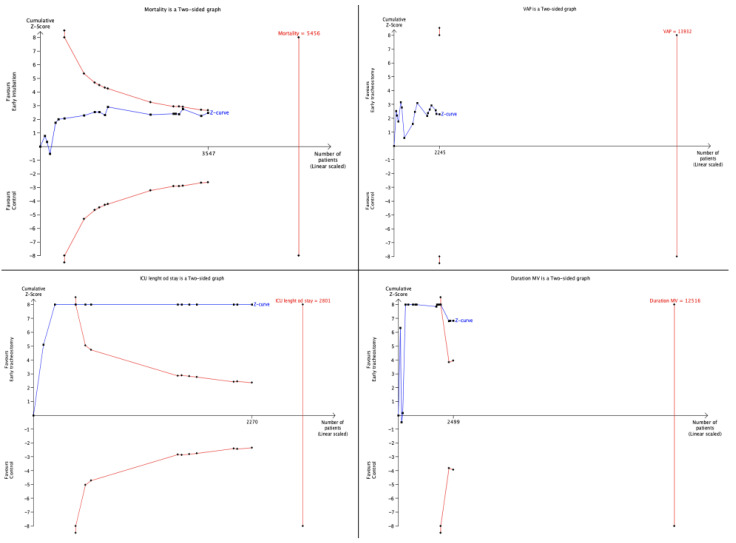
Trial sequential analysis performed for each considered outcome.

The TSA for mortality-adjusted 95% CI ranged from 0.71 to 0.96 (Figure 7). The cumulative Z-curve crossed neither the conventional boundary for benefit nor the trial sequential futility boundary for benefit, suggesting that the current evidence was inconclusive. The TSA for VAP-adjusted 95% CI ranged from 0.64 to 0.97. The cumulative Z-curve crossed neither the conventional boundary for benefit nor the trial sequential futility boundary for benefit, suggesting that the current evidence was inconclusive. TSA for ICU length of stay-adjusted 95% CI ranged from −8.08 to −6.06. TSA for ICU length of stay showed that the line of cumulative *Z*-curve crossed the conventional boundary for benefit, but not the lines of the trial sequential monitoring boundary for benefit and required information size (RIS), which established that evidence was inconclusive and suggests that further trials are needed. TSA for duration of MV-adjusted 95% CI ranged from −3.60 to −1.99. TSA for duration of MV showing that the line of cumulative *Z*-curve crossed the conventional boundary for benefit, but not the lines of the trial sequential monitoring boundary for benefit and required information size (RIS), which established that evidence was inconclusive and suggests that further trials are needed.

## 4. Discussion

To date, there is still much debate about the impact of early tracheostomy on outcomes in critically ill patients. Several RCTs have been conducted on this topic with conflicting results. The same has emerged from recent meta-analyses. From this systematic review with meta-analysis, it emerged that (1) early tracheostomy slightly reduced mortality compared with control, although these results were not confirmed in subgroup analyses, (2) early tracheostomy reduced ICU length of stay and duration of mechanical ventilation compared with late tracheostomy; however, early tracheostomy did not reduce the duration of mechanical ventilation compared with prolonged intubation, and (3) early tracheostomy did not affect VAP. In addition, the TSA performed for each outcome considered did not find conclusive evidence, suggesting the need for further studies.

These findings are novel compared with those from previous systematic reviews and meta-analyses that evaluated the effects of early versus late tracheostomy in critically ill patients undergoing mechanical ventilation. In fact, our analysis showed early tracheostomy reduced mortality compared with the control group but, interestingly and innovatively, this result was not confirmed in subgroup analyses in which early tracheostomy was compared with late tracheostomy and prolonged intubation. Previous meta-analyses produced conflicting results regarding mortality. Shan et al. conducted a meta-analysis of six RCTs and five observational studies and found that early tracheostomy was associated with significant reductions in mortality. However, these results were variable with pools of RCT or non-RCT studies, suggesting that the findings should be interpreted with caution [34]. In another meta-analysis of nine RCTs, Huang et al. found that early tracheostomy did not significantly reduce short- or long-term mortality [35]. A recent meta-analysis based on 15 RCTs concluded that early tracheostomy does not reduce the risk of short-term mortality [18].

In our meta-analysis, early tracheostomy reduced the length of ICU stay and the duration of mechanical ventilation compared with late tracheostomy but did not reduce the duration of mechanical ventilation compared with prolonged intubation. The results of previous meta-analyses are also conflicting for this outcome. Liu et al., in a meta-analysis of 11 RCTs, found that early tracheostomy performed within 7 days of intubation can reduce the length of ICU stay; no significant differences in the duration of mechanical ventilation were observed between groups [16]. Meng et al. conducted a meta-analysis of nine RCTs and found that early tracheostomy may be able to reduce the duration of sedation but did not significantly alter the duration of mechanical ventilation and the length of ICU stay [15]. Another meta-analysis of 12 RCTs concluded that early tracheostomy was associated with more ventilator-free days and a shorter intensive care unit stay than late tracheostomy [36].

As mentioned above, early tracheostomy did not reduce the duration of mechanical ventilation compared to prolonged intubation in our analysis. Four RCTs included in the meta-analysis evaluated the effects of early tracheostomy versus prolonged intubation on critically ill patients undergoing mechanical ventilation [9,22,24,29]. Three of these studies found no difference in the duration of mechanical ventilation [9,24,29], whereas Bouderka et al. found that early tracheostomy was associated with a shorter duration of mechanical ventilation than prolonged intubation [22].

In this meta-analysis, we found that early tracheostomy did not affect VAP. Again, previous meta-analyses have produced conflicting results. A meta-analysis of 14 RCTs concluded that early tracheostomy (within 10 days) was not associated with any difference in the incidence of VAP [37].

A meta-analysis conducted by Liu et al. included 11 RCTs and found that early tracheostomy did not reduce the VAP incidence [38]. Chorath et al. performed a meta-analysis of 17 RCTs and found that, compared with late tracheostomy, early tracheostomy was associated with lower VAP rates [39].

Our results should be interpreted with caution because TSA performed for each considered outcome did not find any conclusive evidence suggesting that further trials are needed to confirm the results of the meta-analysis. Particularly, not a single TSA performed for each outcome reached the required sample size. Indeed, we need 1.909 more patients to reach the required sample size for mortality, 11.687 more patients for VAP, 531 more patients for ICU length of stay and 10.017 more patients for duration of mechanical ventilation. According to this, further studies are required to better identify patients requiring tracheostomy in larger trials, stratifying patients according to the underlying conditions and timing of procedures [40,41].

Furthermore, our results must be interpreted considering several limitations. Firstly, there was marked heterogeneity between studies for some outcome measures, probably related to patient groups with a wide range of characteristics, different timing of tracheotomy and the fact that respiratory management may have changed between 1990 and 2022, the publication dates of the included studies. Secondly, the level of heterogeneity was very high for all analyses and the results must therefore be interpreted with caution despite the use of the random-effects model. Third, the analysis is based on published RCT studies and publication bias is unavoidable. Fourth, adverse effects and cost-effectiveness were not assessed. Fifth, among the studies included in the meta-analysis, there was no clarity on the criteria used for the diagnosis of VAP, while as far as LOS is concerned, none of the included studies clarified whether the length of stay in intensive care is related to the needs of the patient or to the limited availability of beds in ordinary wards. Finally, it should be emphasized that only one study on COVID-19 patients was included in our meta-analysis, so it was not possible to perform a subgroup analysis to find out whether early tracheostomy might have a positive effect on outcomes in this subpopulation of patients.

## 5. Conclusions

Early tracheostomy might have, at least, some benefits on all clinical outcomes considered compared to late tracheostomy and prolonged intubation; however, based on the sequential analyses of the conducted trials, these results are far from conclusive. Further studies with large sample sizes are needed to confirm our results. Furthermore, there is a need for these studies to have a clearer definition of timing, diagnostic criteria for VAP and to analyze long-term mortality, as these data are often absent in the studies analyzed in our analysis.

## Figures and Tables

**Table 1:** Baseline characteristics of studies included in the systematic review and meta-analysis.

Study (First Author, Year)	Country	Setting	Timing		Sample Size	
			Early	Control	Early	Control
Rodriguez, 1990 [20]	USA	Surgical ICU	≤7	≥8	51	55
Saffle, 2002 [21]	USA	Burn ICU	2–3	≥14	21	23
Bouderka, 2004 [22]	Morocco	Trauma ICU	5–6	Prolonged intubation	31	31
Rumbak, 2004 [10]	USA	Medical ICU	≤2	14–16	60	60
Barquist, 2006 [23]	USA	Trauma ICU	≤7	≥29	29	31
Blot, 2008 [9]	France	Surgical ICU	≤4	Prolonged intubation	61	62
Terragni, 2010 [11]	Italy	General ICU	6–8	13–15	209	210
Trouillet, 2011 [24]	France	Cardiac ICU	≤5	Prolonged intubation	109	107
Koch, 2012 [25]	Germany	Surgical ICU	≤4	≥6	50	50
Zheng, 2012 [13]	China	Surgical ICU	3	15	58	61
Bosel, 2013 [26]	Germany	Surgical ICU	≤3	7–14	30	30
Young, 2013 [12]	UK	General and cardiac ICU	≤4	≥10	451	448
Diaz-prieto, 2014 [27]	Spain	ICU	<8	>14	245	244
Mohamed, 2014 [28]	Egypt	ICU	<10	≥10	20	20
Filaire, 2014 [29]	France	ICU	1	Prolonged intubation	39	39
Karlovic, 2018 [30]	Bosnia and Herzegovina	ICU	2–4	≥15	38	42
Goo, 2022 [31]	Malaysia	Neurosurgical ICU	<7	≥7	20	19
Bosel, 2022 [32]	USA and Germany	Neurocritical ICU	≤5	≥10	188	194
Olofsson, 2022 [33]	Sweden	ICU	≤7	≥10	72	78

**Table 2:** Risk of bias assessment of included studies.

Study, Year	Random Sequence Generation	Allocation Concealment	Performance Bias	Detection Bias	Attrition Bias	Selective Reporting	Other Bias
Rodriguez, 1990 [20]	High	High	Unclear	Unclear	Low	Low	Unclear
Saffle, 2002 [21]	Low	Low	Unclear	Unclear	Low	Low	Low
Bouderka, 2004 [22]	Low	Low	Unclear	Unclear	Low	Low	Low
Rumbak, 2004 [10]	Low	Low	Low	Unclear	Low	Low	Low
Barquist, 2006 [23]	Low	Low	Unclear	Unclear	Low	Low	High
Blot, 2008 [9]	Low	Low	Low	Unclear	Low	Low	Low
Terragni, 2010 [11]	Low	Low	Low	Low	Low	Low	Low
Trouillet, 2011 [24]	Low	Low	Low	Unclear	Low	Low	Low
Koch, 2012 [25]	Low	Low	Low	Low	Low	Low	Low
Zheng, 2012 [13]	Low	Low	Low	Unclear	Low	Low	Low
Bosel, 2013 [26]	Low	Unclear	Low	Unclear	Low	Low	Low
Young, 2013 [12]	Low	Low	Low	Low	Low	Low	Low
Diaz-Prieto, 2014 [27]	Low	Low	Unclear	Unclear	Low	Low	Low
Mohamed, 2014 [28]	Low	Unclear	High	High	Low	Low	High
Filaire, 2014 [29]	Unclear	Low	Low	Unclear	Low	Low	Low
Karlović, 2018 [30]	Unclear	Low	Low	Unclear	Low	Low	Low
Goo, 2022 [31]	Low	Low	Low	Low	Low	Low	Low
Bosel, 2022 [32]	Low	Low	Low	Low	Low	Low	High
Olofsson, 2022 [33]	Low	Low	Low	Low	Low	Low	Low

## Data Availability

The datasets used and/or analyzed during the current study are available from the corresponding author on reasonable request.

## Supplementary Materials

The following supporting information can be downloaded at: https://www.mdpi.com/article/10.3390/life14091165/s1, References [10,12,13,20,21,22,23,24,25,26,27,28,29,30,31,32,33] are cited in the supplementary materials.
