# Supplementary material for: Timing of Tracheostomy in ICU Patients: A Systematic Review and Meta-Analysis of Randomized Controlled Trials
Source: Life (Basel). 2024 Sep 14;14(9):1165. doi: 10.3390/life14091165 (PMC11433256; doi:10.3390/life14091165)
Supplement: Supplementary file 1 [file life-14-01165-s001.zip › life-3186398-supplementary.pdf]

Heterogeneity statistics, funnel plot and publication bias for mortality early vs control

Heterogeneity Statistics

Tau	Tau²	I²	H²	R²	df	Q	p
0.167	0.0278 (SE= 0.0296)	34.46%	1.526	.	17.000	25.937	0.076

Model Fit Statistics and Information Criteria

	log-likelihood	Deviance	AIC	BIC	AICc
Maximum-Likelihood	−7.429	24.956	18.858	20.639	19.658
Restricted Maximum-Likelihood	−7.676	15.351	19.351	21.018	20.208

Funnel Plot

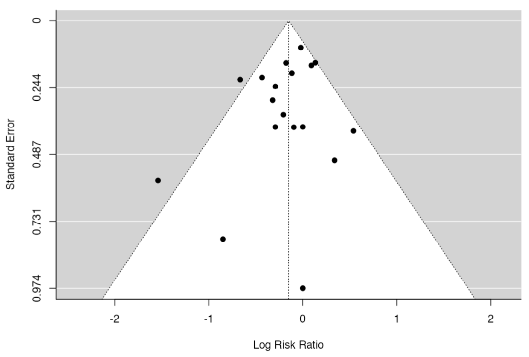

Publication Bias Assessment

Fail-Safe N Analysis (File Drawer Analysis)

Fail-safe N	p
30.000	0.004

Nota. Fail-safe N Calculation Using the Rosenthal Approach

Rank Correlation Test for Funnel Plot Asymmetry

Kendall's Tau	p
−0.085	0.654

Regression Test for Funnel Plot Asymmetry

Z	p
−1.018	0.309

A total of k=18 studies were included in the analysis. The observed log risk ratios ranged from -1.5404 to 0.5390, with the majority of estimates being negative (67%). The estimated average log risk ratio based on the random-effects model was \hat{\mu} = -0.1511 (95% CI: -0.2951 to -0.0070). Therefore, the average outcome differed significantly from zero (z = -2.0555, p = 0.0398). The Q-test for heterogeneity was not significant, but some heterogeneity may still be present in the true outcomes (Q(17) = 25.9367, p = 0.0756, tau^2 = 0.0278, I^2 = 34.4559%). A 95% prediction interval for the true outcomes is given by -0.5084 to 0.2063. Hence, although the average outcome is estimated to be negative, in some studies the true outcome may in fact be positive. An examination of the studentized residuals revealed that none of the studies had a value larger than ± 2.9913 and hence there was no indication of outliers in the context of this model. According to the Cook's distances, none of the studies could be considered to be overly influential. Neither the rank correlation nor the regression test indicated any funnel plot asymmetry (p = 0.6540 and p = 0.3085, respectively).

Forest plot for Early vs late

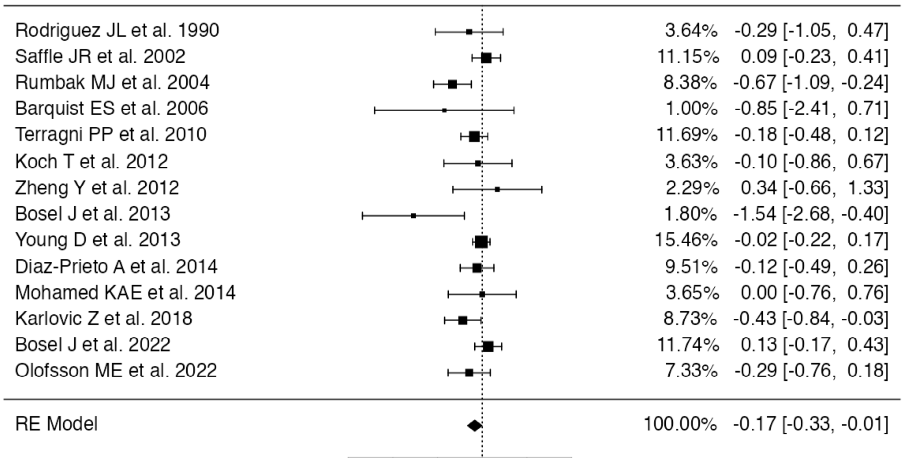

Random-Effects Model (k = 14)

	Estimate	se	Z	p	CI Lower Bound	CI Upper Bound
Intercept	−0.167	0.0818	−2.04	0.041	−0.327	−0.007
	.	.	.	.	.	.

Nota. Tau² Estimator: DerSimonian-Laird

The analysis was carried out using the log risk ratio as the outcome measure. A random-effects model was fitted to the data. The amount of heterogeneity (i.e., tau²), was estimated using the DerSimonian-Laird estimator (Dersimonian 1986). In addition to the estimate of tau², the Q-test for heterogeneity (Cochran 1954) and the I² statistic are reported. In case any amount of heterogeneity is detected (i.e., tau² > 0, regardless of the results of the Q-test), a prediction interval for the true outcomes is also provided. Studentized residuals and Cook's distances are used to examine whether studies may be outliers and/or influential in the context of the model. Studies with a studentized residual larger than the 100 x (1 - 0.05/(2 X k))th percentile of a standard normal distribution are considered potential outliers (i.e., using a Bonferroni correction with two-sided alpha = 0.05 for k studies included in the meta-analysis). Studies with a Cook's distance larger than the median plus six times the interquartile range of the Cook's distances are considered to be influential. The rank correlation test and the regression test, using the standard error of the observed outcomes as predictor, are used to check for funnel plot asymmetry.

A total of k=14 studies were included in the analysis. The observed log risk ratios ranged from -1.5404 to 0.3381, with the majority of estimates being negative (71%). The estimated average log risk ratio based on the random-effects model was \hat{\mu} = -0.1670 (95% CI: -0.3273 to -0.0067). Therefore, the average outcome differed significantly from zero (z = -2.0424, p = 0.0411). According to the Q-test, the true outcomes appear to be heterogeneous (Q(13) = 22.6967, p = 0.0455, tau² = 0.0334, I² = 42.7230%). A 95% prediction interval for the true outcomes is given by -0.5596 to 0.2255. Hence, although the average outcome is estimated to be negative, in some studies the true outcome may in fact be positive. An examination of the studentized residuals revealed that none of the studies had a value larger than ± 2.9137 and hence there was no indication of outliers in the context of this model. According to the Cook's distances, none of the studies could be considered

to be overly influential. Neither the rank correlation nor the regression test indicated any funnel plot asymmetry (p = 0.1572 and p = 0.0960, respectively).

Heterogeneity statistics, funnel plot and publication bias for mortality early vs late

Heterogeneity Statistics

Tau	Tau²	I²	H²	R²	df	Q	p
0.183	0.0334 (SE= 0.0332)	42.72%	1.746	.	13.000	22.697	0.045

Model Fit Statistics and Information Criteria

	log-likelihood	Deviance	AIC	BIC	AICc
Maximum-Likelihood	−5.281	21.515	14.561	15.839	15.652
Restricted Maximum-Likelihood	−5.546	11.092	15.092	16.222	16.292

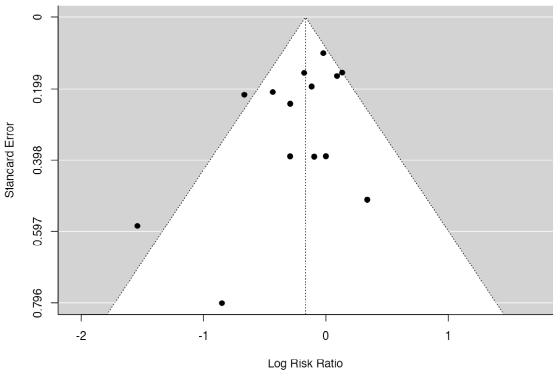

Publication Bias Assessment

Fail-Safe N Analysis (File Drawer Analysis)

Fail-safe N	p
31.000	0.002

Nota. Fail-safe N Calculation Using the Rosenthal Approach

Rank Correlation Test for Funnel Plot Asymmetry

Kendall's Tau	p
−0.297	0.157

Regression Test for Funnel Plot Asymmetry

Z	p
−1.665	0.096

Forest plot for Early vs prolonged intubation

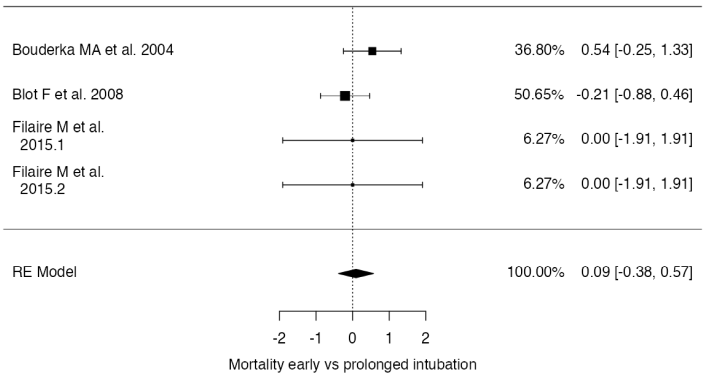

Random-Effects Model (k = 4)

	Estimate	se	Z	p	CI Lower Bound	CI Upper Bound
Intercept	0.0936	0.244	0.384	0.701	−0.385	0.572
	.	.	.	.	.	.

Nota. Tau² Estimator: DerSimonian-Laird

The analysis was carried out using the log risk ratio as the outcome measure. A random-effects model was fitted to the data. The amount of heterogeneity (i.e., tau^2), was estimated using the DerSimonian-Laird estimator (Dersimonian 1986). In addition to the estimate of tau^2, the Q-test for heterogeneity (Cochran 1954) and the I^2 statistic are reported. In case any amount of heterogeneity is detected (i.e., tau^2 > 0, regardless of the results of the Q-test), a prediction interval for the true outcomes is also provided. Studentized residuals and Cook's distances are used to examine whether studies may be outliers and/or influential in the context of the model. Studies with a studentized residual larger than the 100 x (1 - 0.05/(2 X k))th

percentile of a standard normal distribution are considered potential outliers (i.e., using a Bonferroni correction with two-sided alpha = 0.05 for k studies included in the meta-analysis). Studies with a Cook's distance larger than the median plus six times the interquartile range of the Cook's distances are considered to be influential. The rank correlation test and the regression test, using the standard error of the observed outcomes as predictor, are used to check for funnel plot asymmetry.

Heterogeneity statistics, funnel plot and publication bias for mortality early vs prolonged intubation

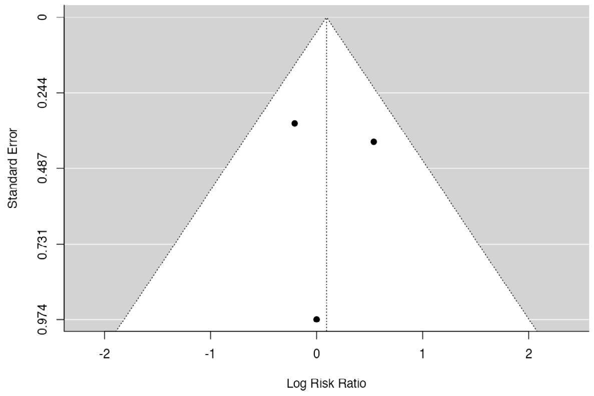

Publication Bias Assessment

Fail-Safe N Analysis (File Drawer Analysis)

Fail-safe N	p
0.000	0.356

Nota. Fail-safe N Calculation Using the Rosenthal Approach

Rank Correlation Test for Funnel Plot Asymmetry

Kendall's Tau	p
0.200	0.702

Regression Test for Funnel Plot Asymmetry

Z	p
0.044	0.965

Heterogeneity statistics, funnel plot and publication bias for VAP early vs control

Heterogeneity Statistics

Tau	Tau²	I²	H²	R²	df	Q	p
0.000	0 (SE= 0.2429)	0%	1.000	.	3.000	2.014	0.569

Model Fit Statistics and Information Criteria

	log-likelihood	Deviance	AIC	BIC	AICc
Maximum-Likelihood	−2.648	2.014	9.297	8.069	21.297
Restricted Maximum-Likelihood	−2.447	4.894	8.894	7.091	20.894

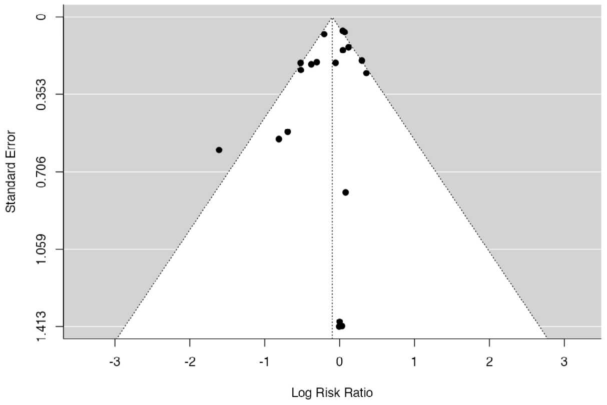

Publication Bias Assessment

Fail-Safe N Analysis (File Drawer Analysis)

Fail-safe N	p
21.000	0.009

Nota. Fail-safe N Calculation Using the Rosenthal Approach

Rank Correlation Test for Funnel Plot Asymmetry

Kendall's Tau	p
−0.181	0.298

Regression Test for Funnel Plot Asymmetry

Z	p
−1.744	0.081

Forest plot for Early vs late

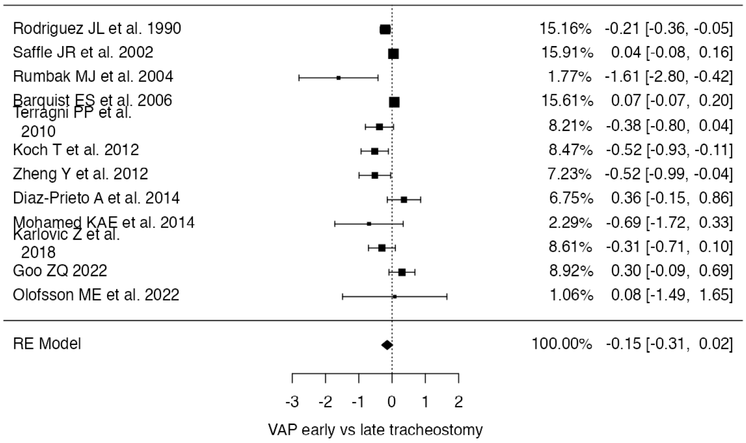

Random-Effects Model (k = 12)

	Estimate	se	Z	p	CI Lower Bound	CI Upper Bound
Intercept	−0.146	0.0851	−1.72	0.086	−0.313	0.021
	.	.	.	.	.	.

Nota. Tau² Estimator: DerSimonian-Laird

The analysis was carried out using the log risk ratio as the outcome measure. A random-effects model was fitted to the data. The amount of heterogeneity (i.e., tau^2), was estimated using the DerSimonian-Laird estimator (Dersimonian 1986). In addition to the estimate of tau^2, the Q-test for heterogeneity (Cochran 1954) and the I^2 statistic are reported. In case any amount of heterogeneity is detected (i.e., $tau^2 > 0$, regardless of the results of the Q-test), a prediction interval for the true outcomes is also provided. Studentized residuals and Cook's distances are used to examine whether studies may be outliers and/or influential in the context of the model. Studies with a studentized residual larger than the 100 x (1 - 0.05/(2 X k))th percentile of a standard normal distribution are considered potential outliers (i.e., using a Bonferroni correction with two-sided alpha = 0.05 for k studies included in the meta-analysis). Studies with a Cook's distance larger than the median plus six times the interquartile range of the Cook's distances are considered to be influential. The rank correlation test and the regression test, using the standard error of the observed outcomes as predictor, are used to check for funnel plot asymmetry.

A total of k=12 studies were included in the analysis. The observed log risk ratios ranged from -1.6094 to 0.3569, with the majority of estimates being negative (58%). The estimated average log risk ratio based on the random-effects model was \hat{\mu} = -0.1461 (95% CI: -0.3129 to 0.0208). Therefore, the average outcome did not differ significantly from zero (z = -1.7158, p = 0.0862). According to the Q-test, the true outcomes appear to be heterogeneous (Q(11) = 35.4719, p = 0.0002, $tau^2 = 0.0417$, $I^2 = 68.9895\%$). A 95% prediction interval for the true outcomes is given by -0.5799 to 0.2877. Hence, although the average outcome is estimated to be negative, in some studies the true outcome may in fact be positive. An examination of the studentized residuals revealed that none of the studies had a value larger than ± 2.8653 and hence there was no indication of outliers in the context of this model. According to the Cook's distances, none of the studies could be considered to be overly influential. Neither the rank correlation nor the

regression test indicated any funnel plot asymmetry (p = 0.2496 and p = 0.0503, respectively).

Heterogeneity statistics, funnel plot and publication bias for VAP early vs late

Heterogeneity Statistics

Tau	Tau²	I²	H²	R²	df	Q	p
0.204	0.0417 (SE= 0.0362)	68.99%	3.225	.	11.000	35.472	< .001

Model Fit Statistics and Information Criteria

	log-likelihood	Deviance	AIC	BIC	AICc
Maximum-Likelihood	−6.409	27.680	16.817	17.787	18.151
Restricted Maximum-Likelihood	−6.711	13.422	17.422	18.217	18.922

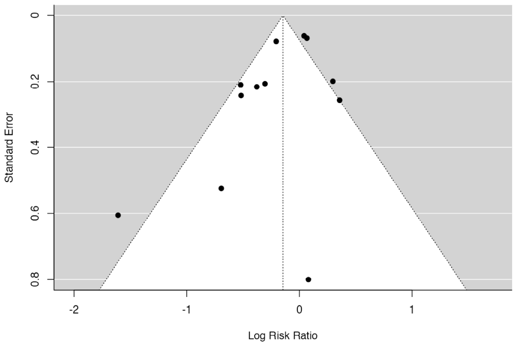

Publication Bias Assessment

Fail-Safe N Analysis (File Drawer Analysis)

Fail-safe N	p
24.000	0.002

Nota. Fail-safe N Calculation Using the Rosenthal Approach

Rank Correlation Test for Funnel Plot Asymmetry

Kendall's Tau	p
−0.273	0.250

Regression Test for Funnel Plot Asymmetry

Z	p
−1.957	0.050

Forest plot for Early vs prolonged intubation

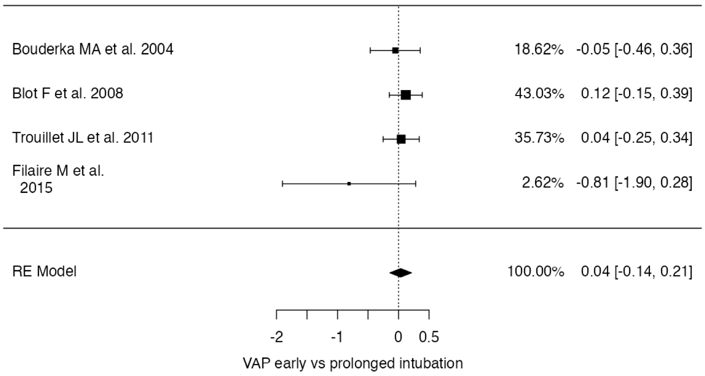

Random-Effects Model (k = 4)

	Estimate	se	Z	p	CI Lower Bound	CI Upper Bound
Intercept	0.0353	0.0902	0.392	0.695	−0.141	0.212

Nota. Tau² Estimator: DerSimonian-Laird

The analysis was carried out using the log risk ratio as the outcome measure. A random-effects model was fitted to the data. The amount of heterogeneity (i.e., tau²), was estimated using the DerSimonian-Laird estimator (Dersimonian 1986). In addition to the estimate of tau², the Q-test for heterogeneity (Cochran 1954) and the I² statistic are reported. In case any amount of heterogeneity is detected (i.e., tau² > 0, regardless of the results of the Q-test), a prediction interval for the true outcomes is also provided. Studentized residuals and Cook's distances are used to examine whether studies may be outliers and/or influential in the context of the model. Studies with a studentized residual larger than the 100 x (1 - 0.05/(2 X k))th

percentile of a standard normal distribution are considered potential outliers (i.e., using a Bonferroni correction with two-sided alpha = 0.05 for k studies included in the meta-analysis). Studies with a Cook's distance larger than the median plus six times the interquartile range of the Cook's distances are considered to be influential. The rank correlation test and the regression test, using the standard error of the observed outcomes as predictor, are used to check for funnel plot asymmetry.

A total of k=4 studies were included in the analysis. The observed log risk ratios ranged from -0.8109 to 0.1189, with the majority of estimates being negative (50%). The estimated average log risk ratio based on the random-effects model was \hat{\mu} = 0.0353 (95% CI: -0.1414 to 0.2120). Therefore, the average outcome did not differ significantly from zero (z = 0.3916, p = 0.6953). According to the Q-test, there was no significant amount of heterogeneity in the true outcomes (Q(3) = 2.8670, p = 0.4126, tau^2 = 0.0000, I^2 = 0.0000%). An examination of the studentized residuals revealed that none of the studies had a value larger than ± 2.4977 and hence there was no indication of outliers in the context of this model. According to the Cook's distances, none of the studies could be considered to be overly influential. Neither the rank correlation nor the regression test indicated any funnel plot asymmetry (p = 0.0833 and p = 0.0934, respectively).

Heterogeneity statistics, funnel plot and publication bias for VAP early vs prolonged intubation

Heterogeneity Statistics

Tau	Tau²	I²	H²	R²	df	Q	p
0.000	0 (SE= 0.0306)	0%	1.000	.	3.000	2.867	0.413

Model Fit Statistics and Information Criteria

	log-likelihood	Deviance	AIC	BIC	AICc
Maximum-Likelihood	0.918	2.867	2.164	0.937	14.164
Restricted Maximum-Likelihood	0.124	–0.248	3.752	1.949	15.752

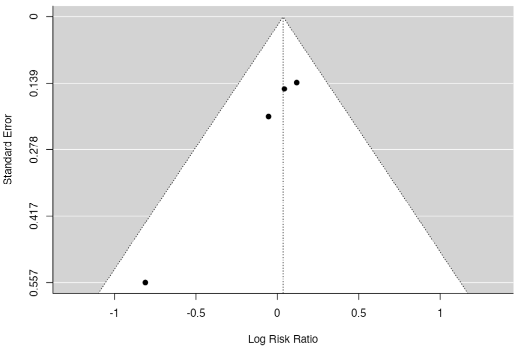

Publication Bias Assessment

Fail-Safe N Analysis (File Drawer Analysis)

Fail-safe N	p
0.000	0.389

Nota. Fail-safe N Calculation Using the Rosenthal Approach

Rank Correlation Test for Funnel Plot Asymmetry

Kendall's Tau	p
–1.000	0.083

Regression Test for Funnel Plot Asymmetry

Z	p
–1.678	0.093

Heterogeneity statistics, funnel plot and publication bias for ICU length of stay early vs control

Heterogeneity Statistics

Tau	Tau²	I²	H²	R²	df	Q	p
0.565	0.3195 (SE= 0.1991)	93.47%	15.315	.	12.000	183.779	<.001

Model Fit Statistics and Information Criteria

	log-likelihood	Deviance	AIC	BIC	AICc
Maximum-Likelihood	−22.789	65.628	49.578	50.708	50.778
Restricted Maximum-Likelihood	−22.372	44.745	48.745	49.714	50.078

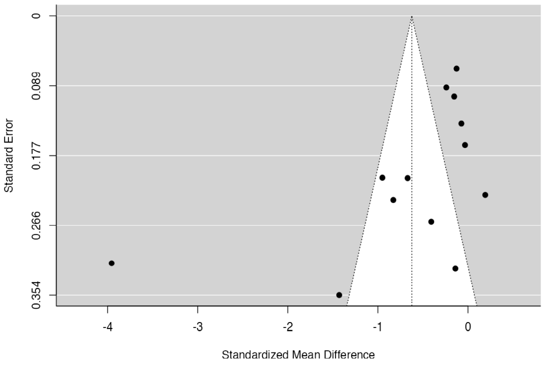

Publication Bias Assessment

Test Name	value	p
Fail-Safe N	467.000	<.001
Begg and Mazumdar Rank Correlation	−0.410	0.057
Egger's Regression	−3.441	<.001
Trim and Fill Number of Studies	0.000	.

Nota. Fail-safe N Calculation Using the Rosenthal Approach

Forest plot for Early vs late

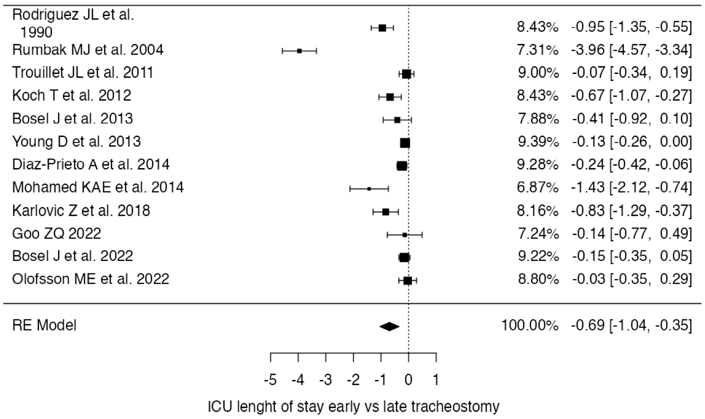

The analysis was carried out using the standardized mean difference as the outcome measure. A random-effects model was fitted to the data. The amount of heterogeneity (i.e., tau^2), was estimated using the DerSimonian-Laird estimator (Dersimonian 1986). In addition to the estimate of tau^2, the Q-test for heterogeneity (Cochran 1954) and the I^2 statistic are reported. In case any amount of heterogeneity is detected (i.e., tau^2 > 0, regardless of the results of the Q-test), a prediction interval for the true outcomes is also provided. Studentized residuals and Cook's distances are used to examine whether studies may be outliers and/or influential in the context of the model. Studies with a studentized residual larger than the 100 x (1 - 0.05/(2 X k))th percentile of a standard normal distribution are considered potential outliers (i.e., using a Bonferroni correction with two-sided alpha = 0.05 for k studies included in the meta-analysis). Studies with a Cook's distance larger than the median plus six times the interquartile range of the Cook's distances are considered to be influential. The rank correlation test and the regression test, using the standard error of the observed outcomes as predictor, are used to check for funnel plot asymmetry.

A total of k=12 studies were included in the analysis. The observed standardized mean differences ranged from -3.9557 to -0.0330, with the majority of estimates being negative (100%). The estimated average standardized mean difference based on the random-effects model was \hat{\mu} = -0.6918 (95% CI: -1.0373 to -0.3462). Therefore, the average outcome differed significantly from zero (z = -3.9237, p < 0.0001). According to the Q-test, the true outcomes appear to be heterogeneous (Q(11) = 179.2880, p < 0.0001, tau^2 = 0.3267, I^2 = 93.8646%). A 95% prediction interval for the true outcomes is given by -1.8641 to 0.4805. Hence, although the average outcome is estimated to be negative, in some studies the true outcome may in fact be positive. An examination of the studentized residuals revealed that one study (Rumbak MJ et al. 2004) had a value larger than ± 2.8653 and may be a potential outlier in the context of this model. According to the Cook's distances, one study (Rumbak MJ et al. 2004) could be considered to be overly influential. Both the rank correlation and the regression test indicated potential funnel plot asymmetry (p = 0.0311 and p = 0.0002, respectively).

Heterogeneity statistics, funnel plot and publication bias for ICU length of stay early vs late

Heterogeneity Statistics

Tau	Tau²	I²	H²	R²	df	Q	p
0.572	0.3267 (SE= 0.2104)	93.86%	16.299	.	11.000	179.288	<.001

Model Fit Statistics and Information Criteria

	log-likelihood	Deviance	AIC	BIC	AICc
Maximum-Likelihood	−21.226	61.375	46.453	47.422	47.786
Restricted Maximum-Likelihood	−20.800	41.601	45.601	46.397	47.101

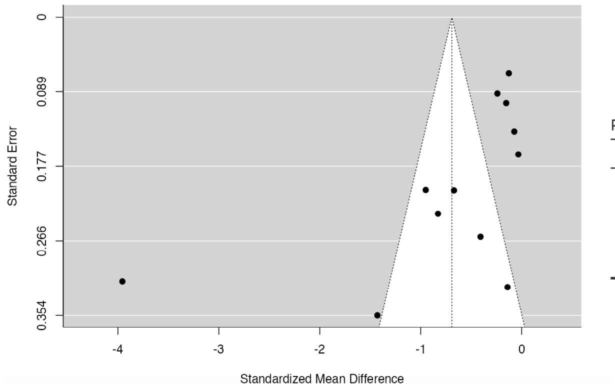

Publication Bias Assessment

Test Name	value	p
Fail-Safe N	491.000	<.001
Begg and Mazumdar Rank Correlation	−0.485	0.031
Egger's Regression	−3.782	<.001
Trim and Fill Number of Studies	0.000	.

Nota. Fail-safe N Calculation Using the Rosenthal Approach

Heterogeneity Statistics

Tau	Tau²	I²	H²	R²	df	Q	p
0.510	0.2605 (SE= 0.1547)	90.17%	10.169	.	14.000	142.364	<.001

Heterogeneity statistics, funnel plot and publication bias for duration of MV early vs control

Model Fit Statistics and Information Criteria

	log-likelihood	Deviance	AIC	BIC	AICc
Maximum-Likelihood	−15.830	52.453	35.659	37.075	36.659
Restricted Maximum-Likelihood	−15.497	30.994	34.994	36.272	36.085

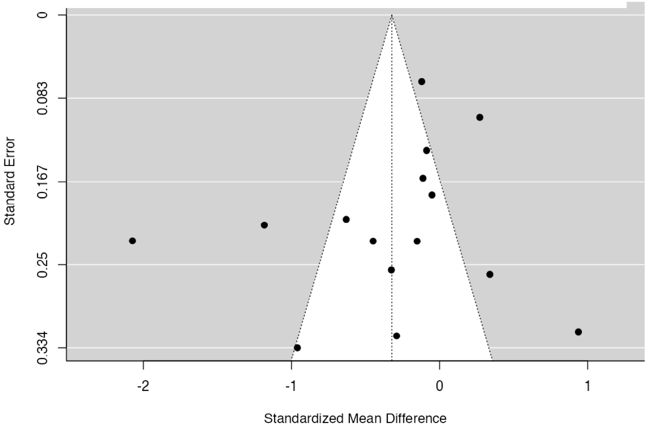

Publication Bias Assessment

Test Name	value	p
Fail-Safe N	166.000	<.001
Begg and Mazumdar Rank Correlation	−0.200	0.328
Egger's Regression	−0.506	0.613
Trim and Fill Number of Studies	0.000	.

Nota. Fail-safe N Calculation Using the Rosenthal Approach

Forest plot for Early vs late

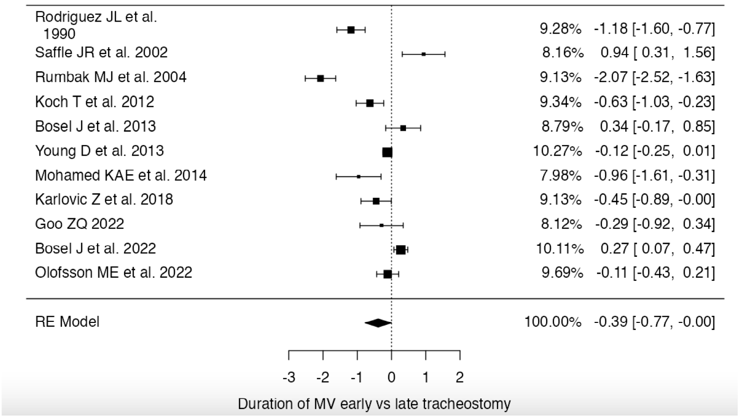

A total of k=11 studies were included in the analysis. The observed standardized mean differences ranged from -2.0739 to 0.9374, with the majority of estimates being negative (73%). The estimated average standardized mean difference based on the random-effects model was \hat{\mu} = -0.3887 (95% CI: -0.7726 to -0.0048). Therefore, the average outcome differed significantly from zero ($z = -1.9845$, $p = 0.0472$). According to the Q-test, the true outcomes appear to be heterogeneous ($Q(10) = 140.9760$, $p < 0.0001$, $tau^2 = 0.3689$, $I^2 = 92.9066\%$). A 95% prediction interval for the true outcomes is given by -1.6395 to 0.8621. Hence, although the average outcome is estimated to be negative, in some studies the true outcome may in fact be positive. An examination of the studentized residuals revealed that one study (Rumbak MJ et al. 2004) had a value larger than ± 2.8376 and may be a potential outlier in the context of this model. According to the Cook's distances, none of the studies could be considered to be overly influential. Neither the rank correlation nor the regression test indicated any funnel plot asymmetry ($p = 0.5423$ and $p = 0.7892$, respectively).

Heterogeneity statistics, funnel plot and publication bias for duration of MV early vs late

Heterogeneity Statistics

Tau	Tau²	I²	H²	R²	df	Q	p
0.607	0.3689 (SE= 0.2605)	92.91%	14.098	.	10.000	140.976	<.001

Model Fit Statistics and Information Criteria

	log-likelihood	Deviance	AIC	BIC	AICc
Maximum-Likelihood	−13.195	41.427	30.390	31.186	31.890
Restricted Maximum-Likelihood	−12.708	25.415	29.415	30.020	31.129

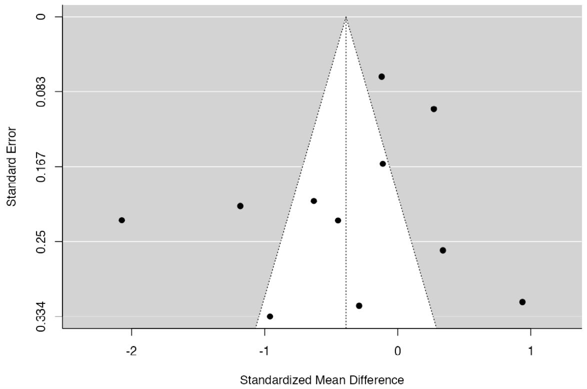

Publication Bias Assessment

Test Name	value	p
Fail-Safe N	126.000	<.001
Begg and Mazumdar Rank Correlation	−0.164	0.542
Egger's Regression	−0.267	0.789
Trim and Fill Number of Studies	0.000	.

Nota. Fail-safe N Calculation Using the Rosenthal Approach

Forest plot for Early vs prolonged

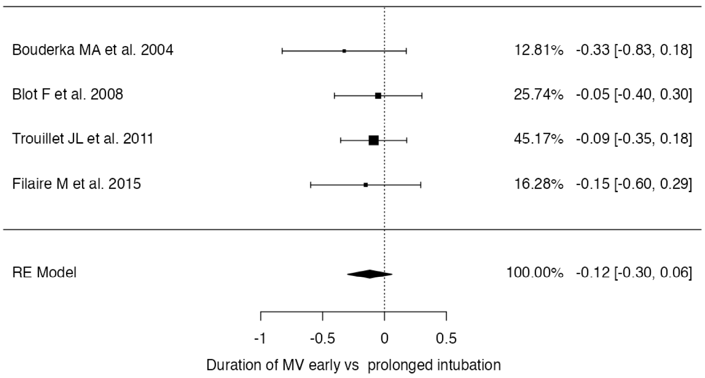

The analysis was carried out using the standardized mean difference as the outcome measure. A random-effects model was fitted to the data. The amount of heterogeneity (i.e., tau²), was estimated using the DerSimonian-Laird estimator (Dersimonian 1986). In addition to the estimate of tau², the Q-test for heterogeneity (Cochran 1954) and the I² statistic are reported. In case any amount of heterogeneity is detected (i.e., tau² > 0, regardless of the results of the Q-test), a prediction interval for the true outcomes is also provided. Studentized residuals and Cook's distances are used to examine whether studies may be outliers and/or influential in the context of the model. Studies with a studentized residual larger than the 100 x (1 - 0.05/(2 X k))th percentile of a standard normal distribution are considered potential outliers (i.e., using a Bonferroni correction with two-sided alpha = 0.05 for k studies included in the meta-analysis). Studies with a Cook's distance larger than the median plus six times the interquartile range of the Cook's distances are considered to be influential. The rank correlation test and the regression test, using the standard error of the observed outcomes as predictor, are used to check for funnel plot asymmetry.

A total of k=4 studies were included in the analysis. The observed standardized mean differences ranged from -0.3255 to -0.0514, with the majority of estimates being negative (100%). The estimated average standardized mean difference based on the random-effects model was \hat{\mu} = -0.1192 (95% CI: -0.2986 to 0.0601). Therefore, the average outcome did not differ significantly from zero (z = -1.3027, p = 0.1927). According to the Q-test, there was no significant amount of heterogeneity in the true outcomes (Q(3) = 0.8670, p = 0.8334, tau^2 = 0.0000, I^2 = 0.0000%). An examination of the studentized residuals revealed that none of the studies had a value larger than ± 2.4977 and hence there was no indication of outliers in the context of this model. According to the Cook's distances, none of the studies could be considered to be overly influential. Neither the rank correlation nor the regression test indicated any funnel plot asymmetry (p = 0.3333 and p = 0.4783, respectively).

Heterogeneity Statistics

Tau	Tau^2	I^2	H^2	R^2	df	Q	p
0.000	0 (SE= 0.0299)	0%	1.000	.	3.000	0.867	0.833

Model Fit Statistics and Information Criteria

	log-likelihood	Deviance	AIC	BIC	AICc
Maximum-Likelihood	2.445	0.867	−0.890	−2.118	11.110
Restricted Maximum-Likelihood	1.666	−3.332	0.668	−1.134	12.668

Heterogeneity statistics, funnel plot and publication bias for duration of MV early vs prolonged intubation

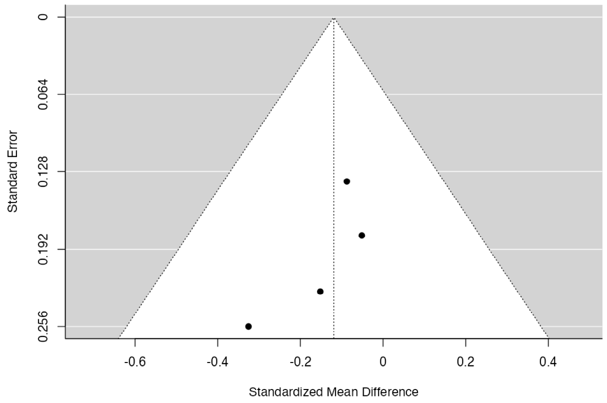

Publication Bias Assessment

Test Name	value	p
Fail-Safe N	0.000	0.076
Begg and Mazumdar Rank Correlation	−0.667	0.333
Egger's Regression	−0.709	0.478
Trim and Fill Number of Studies	1.000	.

Nota. Fail-safe N Calculation Using the Rosenthal Approach
